# The Institutional Library

**Published:** 1915-09-11

**Authors:** 


### THE INSTITUTIONAL LIBRARY.

### A Handbook for Parents.

Towards Racial Health: A Handbook for Parents, Teachers, and Social Workers on the Training of Boys and Girls. By Norah H. March, B.Sc. With a Foreword by J. Arthur Thomson, M.A., LL.D. (London: George Routledge and Sons, Limited. 1915. Pp. 326+viii. Price 3s. 6d. net.)

That the classes to whom this handbook is addressed often feel the need of wise direction in speaking to children on the subject of sex may be readily allowed; and we are more than willing to recognise that Miss March has made a sustained and earnest effort to provide this direction. We venture, however, to doubt whether her method is a wise one, and we question whether the adoption of it would lead to the end she sincerely desires—namely, the growth of healthy mental habits in boys and girls. The principle upon which she rests her method is that "we must begin to unfold the story of birth during the years of little childhood," and after this principle has been grasped "the story of human parenthood" must be introduced into the child's mind, which may thus be prepared for the "dawn of adolescence." To effect this teaching she advocates the contemplation and demonstration of the reproductive processes in the "plants and animals about us." Much of her book is occupied by a description of these processes and of the organs which set them in operation, the object being to enable the teacher to familiarise the child with the universality and importance of sex operations. Even for adults and teachers there seems to us to be in her pages an unnecessary amount of anatomical and physiological detail, and to lead children to regard plants and animals as opportunities for the discovery of sexual organs and activities is surely likely to stimulate a morbid curiosity and a one-sided view of life rather than to produce clean and healthy minds. If the child is to be led to regard poppies, tulips, daffodils, willows, hazels, hops, earth-worms, water-snails, land-snails, slugs, spiders, sticklebacks, toads, sparrows, rabbits, mice, guinea-pigs, cats, dogs—not to speak of others—as constituting "a biological approach to sex enlightenment" and a "route towards matters concerning human sex," we are likely to hear some curious comments from the sexual prigs we shall meet at our family breakfast tables. It may be that the conventional conspiracy of silence on such matters carries risks, but we doubt whether the alternative proposed by Miss March is the pathway of safety. That parents and guardians can be helped in their responsibilities relative to the sex-education of children by a little healthy, robust, and sensible advice we fully agree, but a book of three hundred pages is not necessary for this purpose, neither is it necessary to contemplate the "courtship dance" of the spider or the "psychical fatherhood" of the stickleback. In the severe atmosphere of the scientific laboratory these and such like phenomena are doubtless the objects of calm and unemotional survey, but they are likely to have a disturbing rather than a helpful influence on a young mother anxious for the welfare of her little ones. We are quite sure that Miss March has written her book with the most worthy motives. Whether she will convince many that the "main facts involved in parenthood" should be learned "before ten years of age" and that the "hermaphroditic animals" should be utilised to this end remains to be seen. Professor Thomson, who writes the Foreword, thinks we have not yet "sufficiently consulted the child" on the subject of "education in racial hygiene." This may be, and yet we fancy, so strongly rooted are British custom and prejudice, that any parent who purchases the book for which the Professor stands as sponsor will be careful to keep it under lock and key.

### Tuberculosis Officers and Nurses.

A Practical Manual of Tuberculosis for Nurses. By L. T. Burra, M.D. Oxon. (London: John Bale, Sons and Danielsson, Ltd. Price 2s.)

Handbooks for the instruction and guidance of nurses engaged in the anti-tuberculosis campaign are now having a small boom. One of the most recently published of these works is written by the tuberculosis officer of the county of Buckingham, and is based on a series of lectures given by him to nurses engaged in district work in that county. Inasmuch as the tuberculosis nurse is too often set to deal with individual patients, and is not yet effectively concerned with the public-health aspect of the tuberculosis problem, the scope of Dr. Burra's little book should meet the demand for an instructive guide to the disease, its treatment, and the general principles involved in nursing cases of pulmonary and other forms of tuberculosis. It would, however, not have been out of place if some space had been devoted to the consideration of the larger problems connected with the reduction of tuberculosis in a community. The largest section of the book provides a good account of pulmonary tuberculosis with a large amount of detail relating to medical matters. It is difficult to avoid trespassing on the province of the doctor in describing such a chronic disease as consumption, and in country districts it is almost necessary for the nurse to have an intelligent comprehension of the general course and complications of the disease, if only that thereby she may not be too ready with meddlesome therapeutics. A useful chapter describes briefly the forms of non-pulmonary tuberculosis, and another chapter is devoted solely to the consideration of the use of tuberculin. This latter is one which hardly fits in with the scope of the book. It is full of details which can be but very dimly appreciated by the average nurse and will therefore be of no practical use to her. Dr. Burra does, however, warn nurses not to suggest the use of tuberculin to any patient! Such a highly technical chapter as this one is a waste of space, and such information tends to exalt unduly the mere tinkering with a treatment of individual cases at the expense of preventive measures. At the end of the book there are several pages of common or garden "puffs" referring to the commodities or other matters advertised at either end of the book. These paragraphs, although separated from Dr. Burra's pages, are no ornament to the book, and will in no wise enhance its reputation. One note is not without its humour, for we learn that a certain sanatorium "has been steadily assimilating knowledge" for some years! Dr. Burra is, of course, absolved from all responsibility for these pages, and they have no doubt helped to allow the book to be published at the very moderate price of two shillings, but they could have been omitted with advantage. Nurses will find this manual a handy guide to the treatment and management of tuberculosis, and the complications of the pulmonary form in particular, but it does not pretend to be a guide to the conduct of an anti-tuberculosis campaign.

Diet and Disease in Infancy. By Hector Chas. Cameron, M.D., F.R.C.P. (London: J. and A. Churchill. 1915. Pp. 208. Price 8s. 6d. net.)

The ambition of this book is to deal with the various problems associated with the feeding of infants both in health and in disease. Dr. Cameron has spared no pains to make his effort a success, and he is to be congratulated on the result. He has produced a volume which is both interesting and serviceable, and the attainment of these characters has been secured by careful study, not merely of books and laboratory tests, but also of the central figure of the problem—namely, the disturbed or suffering infant. The adjustment of scientific theory to the practical possibilities of the existing situation is indeed one of the distinctive notes of the book, which equally avoids both the danger of raising the reader to the clouds and leaving him there, and, on the other hand, the harsh dogmatism which carries no illumination. As a brief outline of the field covered, we note the consideration of the conditions necessary for a satisfactory milk supply, the management of breast feeding and artificial feeding, the various forms of infantile dyspepsia, marasmus, inanition, the premature infant, constipation, rickets, etc. All these are discussed in the true clinical spirit and with a consciousness of practical ends. We venture a special word of recognition of the literary quality which distinguishes the book, and which is so grateful to the reader who respects his mother tongue.

An Index of Symptoms with Diagnostic Methods. By Ralph Winnington Leftwich, M.D. Fifth Edition. (London: Smith, Elder and Co. 1915. Pp. 516 + xii. Price 10s. 6d. net.)

Dr. Leftwich's Index has grown from a book for the pocket into a book for the desk. Though some may regret the increase in size, personally we think it to be fully justified. Certainly the work is a monument to the industry and to the keen clinical outlook of its author. As a means of rapidly surveying the possible interpretations which may be attached to any individual symptom it stands without a rival, and its completeness in this respect is little short of marvellous. Many of us would shrink from examination on the nature and meaning of Jaworski's test, or Frimadeau's sign, or Williams' phenomenon, or Pagano's reaction, but all these and a multitude of others are duly explained in the Index. It is no wonder, therefore, that the distressed clinician and the puzzled student have combined to carry Dr. Leftwich's book into a fifth edition. To detect in it anything in the nature of an omission is a difficult task, but when we are told so much of what we were previously ignorant we may perhaps venture to suggest that mitral stenosis has been noted with sufficient frequency as a "cause" of paralysis of the recurrent laryngeal nerve to merit inclusion in the author's list. Necessarily the definitions which are afforded have to be brief, and they are therefore in some instances wanting in completeness. Thus it is hardly sufficient to say of pulsus alternans that the "beats are unequal in strength," or of hemiopia that it is due to a "lesion in the posterior portion of the internal capsule," seeing that other anatomical situations are at least possible. The representations of pulse tracings, we think, could be improved, as, for example, in the case of aortic stenosis, where the "typical sphygmogram" fails to illustrate the definition that "the tidal wave is stronger than the summit wave." In its general purpose and achievement, however, the book is beyond praise, and the sustained care and pains necessary to maintain it at the level of present-day knowledge are a proof of diligence which we may envy though we can hardly hope to emulate it.

Amœbiasis and the Dysenteries. By L. P. Phillips, M.A., M.D., B.C. (Cantab.), etc. (London: H. K. Lewis. 1915. Pp. 147 + xi. Price 6s. 6d. net.)

The practical value of thoroughness of investigation into the causes of disease and the establishment of these upon the basis of confident knowledge could hardly be better illustrated than by the relatively recent history of the treatment of dysentery. Not so long ago this name merely defined a certain clinical association of symptoms, and all cases fell under a common classification and received inevitably the same order of therapeutic discipline. Yet to-day it can be laid down with great confidence that while in amœbic dysentery emetine is a definitely specific remedy, this drug has no action in the bacillary form of the disease, which, on the contrary, needs the influence of a specific antitoxin. In other words, cases formerly arranged in one and the same group can now be surely separated, and each may receive its appropriate remedy. How this and allied movements have come about is one of the most fascinating pages in medical history. Professor Phillips has a very wide knowledge of the subject, and his book gives a complete description of the story of amœbic infection. Interesting in itself, it has a special value at this time of almost universal war. Returning soldiers may possibly introduce the disease into this country, and in this respect it is specially noteworthy that an individual may be a carrier of the amœbæ and a spreader of the disease, though he himself has never been a victim. Hence there is good reason why we at home should keep in mind the possible development of dysentery and of its various complications. No better authority can be consulted than the book now before us, and we cordially commend it to the attention of our readers.

Materia Medica and Pharmacy. By Reginald R. Bennett, B.Sc. Lond., F.I.C. (London: H. K. Lewis. 1915. Pp. 242 + xxiv. Price 4s. 6d. net.)

Mr. Bennett's book is intended for the use of medical students. It recognises that the needs of these in reference to materia medica and pharmacy are different from the requirements of pharmaceutical students. To prescribe medicines is one thing, to dispense them is another. That there is a certain area of knowledge which is common to both departments is evident, but to insist, as do some writers, on the physical properties of medicines and on manufacturing processes is to ask too much from the medical student. These considerations are kept at a minimum in Mr. Bennett's pages. The medical practitioner certainly ought to know medicines as things and not merely as names, but he cannot be expected to judge their commercial value or to appreciate their microscopic characters. We quite agree that a practical course at a dispensing counter is of great value to a medical student, and we regret that practical pharmacy occupies so careless a position in the medical curriculum. Mr. Bennett's book will not atone for this defect, but its plan is excellent, and the execution throughout is marked by great care. An attempt is made to classify the various remedies according to their therapeutic actions. This is helpful to the student, but is not in all respects satisfactory. It gives a certain measure of interest to a subject which is proverbially "dry," and hence may be regarded as justified in the circumstances. The "tables" of doses, standard strengths, and so on are very well arranged and will be welcome both for reference and for examination purposes.

